# Gut microbiota in colorectal cancer: a review of its influence on tumor immune surveillance and therapeutic response

**DOI:** 10.3389/fonc.2025.1557959

**Published:** 2025-03-05

**Authors:** Chunlei Zhang, Yong Wang, Lei Cheng, Xiansheng Cao, Chunyuan Liu

**Affiliations:** ^1^ Department of Colorectal and Anus Surgery, Yantai Affiliated Hospital of Binzhou Medical University, Yantai, China; ^2^ Department of Hepatobiliary Surgery, Yantai Affiliated Hospital of Binzhou Medical University, Yantai, China; ^3^ Department of Gastrointestinal Surgery, Hernia and Abdominal Wall Surgery I, Yantai Affiliated Hospital of Binzhou Medical University, Yantai, China

**Keywords:** colorectal cancer, gut microbiota, tumor immune surveillance, therapeutic response, probiotics, prebiotics, fecal microbiota transplantation

## Abstract

Colorectal cancer (CRC) poses a significant global health burden, with gut microbiota emerging as a crucial modulator of CRC pathogenesis and therapeutic outcomes. This review synthesizes current evidence on the influence of gut microbiota on tumor immune surveillance and responses to immunotherapies and chemotherapy in CRC. We highlight the role of specific microbial taxa in promoting or inhibiting tumor growth and the potential of microbiota-based biomarkers for predicting treatment efficacy. The review also discusses the implications of microbiota modulation strategies, including diet, probiotics, and fecal microbiota transplantation, for personalized CRC management. By critically evaluating the literature, we aim to provide a comprehensive understanding of the gut microbiota’s dual role in CRC and to inform future research directions in this field.

## 1 Introduction

Colorectal cancer (CRC) remains a leading cause of cancer-related mortality worldwide, accounting for approximately 10% of all cancer deaths, according to the Global Cancer Observatory (GLOBOCAN 2020) (1). The American Cancer Society estimated that there were about 151,030 new cases of CRC and approximately 52,580 deaths in the United States in 2022 alone (2). Risk factors contributing to the rise of CRC include genetic predispositions, dietary habits, sedentary lifestyles, and the increasing role of alterations in gut microbiota (3, 4). Notably, advancements in our understanding of tumor biology have illuminated the role of the gut microbiome in immune system modulation and its implications for tumor surveillance and therapeutic responses in CRC (5).

The gut microbiota, which is composed of trillions of microorganisms, including bacteria, viruses, fungi, and archaea, plays essential roles in metabolism, vitamin synthesis, and maintaining immune homeostasis (6). Recent studies have underscored the impact of dysbiosis—imbalance in the composition of gut microbiota—on CRC pathogenesis and progression (7). Research highlights differences in microbial composition between CRC patients and healthy individuals, suggesting that specific gut microbial patterns might serve as both biomarkers for early detection and targets for novel therapeutic strategies (8).

The relationship between gut microbiota and CRC extends beyond mere correlation. Experimental models of CRC have demonstrated that certain bacterial species, such as Fusobacterium nucleatum, can promote tumor growth through pro-inflammatory mechanisms and immune evasion, while beneficial species, such as *Lactobacillus*, might exert protective effects (9, 10). These observations raise critical questions regarding the mechanisms by which gut microbiota modulate both tumor immunity and responsiveness to therapies, particularly immune checkpoint inhibitors and chemotherapy.

Recent investigations suggest a substantial impact of gut microbiota on immune checkpoint blockade efficacy in CRC. For instance, researchers have shown that the presence of specific microbial taxa correlates with enhanced response rates to programmed death factor-1(PD-1) inhibitors (11). Conversely, other studies indicate that certain gut microbial profiles could render tumors resistant to immunotherapy, calling for a more nuanced understanding of microbiota-related mechanisms, which could facilitate the personalized treatment of CRC (12).

However, heterogeneity in study results has led to debates regarding the specific microbial species involved, the methodologies employed in microbiome profiling, and factors such as host genetics and diet that may contribute to these differences (13). For example, while *Bacteroides* fragilis has been implicated in tumorigenesis in some populations, it has shown protective effects in others, emphasizing the complexity of host-microbe interactions (13). Additionally, the usage of various sequencing techniques and bioinformatics tools adds layers of variability that challenge the reproducibility of findings across studies.

In this review, we will systematically evaluate existing literature focused on gut microbiota’s role in CRC, emphasizing its influence on tumor immune surveillance and therapeutic responses. By critically analyzing different studies and their outcomes, we aim to provide a comprehensive overview of the current understanding, along with highlighting gaps and inconsistencies in the research that warrant further investigation. The goal is to refine our perspective on gut microbiota’s dual role as both a biological entity influencing tumorigenesis and a potential therapeutic target, fostering future research endeavors in CRC prevention and treatment.

## 2 The role of gut microbiota in CRC

**Figure 1:**
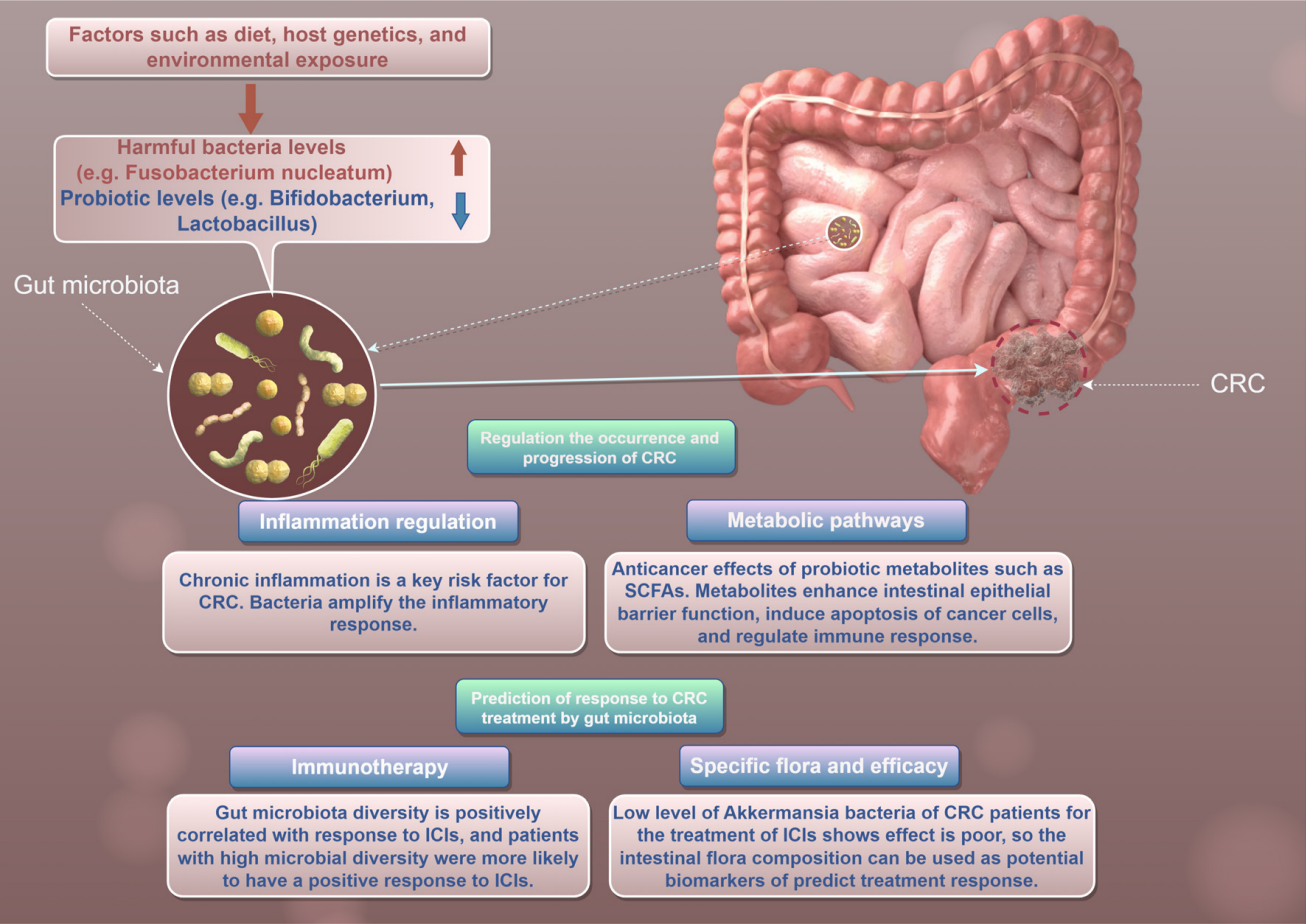
This figure to show the role of gut microbiota in CRC by Figdraw.

CRC is one of the leading causes of cancer-related morbidity and mortality worldwide (14). Emerging evidence suggests that gut microbiota plays a crucial role in the pathogenesis and progression of CRC (15) (**Figure 1**). The human gut is home to trillions of microorganisms, collectively referred to as the gut microbiota, which are involved in various physiological processes (6). Recent studies indicate that alterations in gut microbial communities may influence tumor immune surveillance and therapeutic responses in CRC patients (16).

### 2.1 Gut microbiota composition in CRC

The composition of gut microbiota can vary significantly between healthy individuals and CRC patients (17). Several studies have reported distinct microbial signatures associated with CRC. For instance, Zhu et al. (9) demonstrated that CRC patients exhibited increased levels of invasive bacteria, such as *Fusobacterium nucleatum*, which have been linked to tumor progression. This bacterium can enhance tumorigenesis through mechanisms such as the promotion of inflammatory responses and modulation of immune cell activities. Conversely, commensal bacteria such as Bifidobacterium may inhibit CRC progression through anti-inflammatory metabolites and immune modulation (18).

Notably, the relationship between gut microbiota and CRC is complex and influenced by various factors, including diet, host genetics, and environmental exposures (19) (**Figure 1**). Environmental factors, such as diet and microbial exposure in the living environment, play a crucial role in shaping the gut microbiota and its impact on CRC. Different dietary patterns can significantly alter the composition of intestinal microbiota, thereby affecting the occurrence of tumors and therapeutic outcomes.

Dietary patterns, such as high-fat diets, have been shown to increase the abundance of pro-inflammatory bacteria and reduce the diversity of the gut microbiota, which may promote tumor development. In contrast, high-fiber diets can enrich beneficial bacteria, such as Bifidobacterium and Lactobacillus, which are associated with a lower risk of CRC (20). These beneficial bacteria can enhance the production of short-chain fatty acids (SCFAs), which have anti-inflammatory and anti-tumor effects (21).

Moreover, microbial exposure in the living environment can also influence the gut microbiota. For example, exposure to certain bacteria or viruses may alter the gut microbiota composition, leading to an increased risk of CRC (22). Understanding these environmental influences can help enrich the research dimension and provide new insights into the prevention and treatment of CRC.

In a meta-analysis by Wirbel et al., the authors highlighted significant disparities in microbial profiles across different studies, emphasizing the need for standardized methodologies in gut microbiota research (23). Factors contributing to these inconsistencies include population diversity, geographic variations, and methodological differences in sampling and analysis. Future research should focus on exploring the complex interplay between environmental factors, gut microbiota, and CRC to develop more effective prevention and treatment strategies.

### 2.2 Mechanisms of gut microbiota in CRC pathogenesis

Gut microbiota’s influence on CRC involves multiple mechanisms, including the modulation of inflammation, metabolic pathways, and tumor immune surveillance (24). One of the key pathways through which gut microbiota contribute to CRC development is by promoting chronic inflammation (24). Chronic inflammation is a well-known risk factor for CRC, with cytokines and other pro-inflammatory mediators being implicated in tumorigenesis (25) (**Figure 1**). For example, *Fusobacterium nucleatum* can amplify the inflammatory response and activate oncogenic signaling pathways, such as the Wnt/β-catenin pathway, leading to increased cell proliferation and survival (26).

Moreover, certain microbial metabolites, particularly SCFAs produced by beneficial gut bacteria, have been shown to exert protective effects against CRC (27). SCFAs, such as butyrate, can enhance intestinal epithelial barrier function, promote apoptosis in cancerous cells, and regulate immune responses (28). According to a study by Kang et al, the oral administration of butyrate significantly reduced tumor incidence in mouse models of CRC, supporting its potential as a therapeutic strategy (29).

Recent studies have further elaborated on the key signaling pathways and molecular targets through which gut microbiota affect tumor immunity and therapeutic response (30, 31). For instance, Fusobacterium nucleatum has been shown to modulate the tumor microenvironment by inducing the expression of pro-inflammatory cytokines such as IL-6, IL-17, and tumor necrosis factor α, which promote angiogenesis and tumor cell metastasis (30). Additionally, Bacteroides fragilis, another bacterium associated with CRC, produces a toxin (BFT) that disrupts cell junctions of E-cadherin and β-catenin, leading to increased intestinal permeability and inflammation (32). BFT exerts its neoplastic effects by causing DNA damage and accumulating mutations, leading to cell proliferation and transcription of genes involved in tumor progression (c-myc) (33, 34).

The Wnt/β-catenin signaling pathway is a key oncogenic pathway that is often activated in CRC. Fusobacterium nucleatum can activate this pathway, leading to increased cell proliferation and survival (35, 36). Furthermore, the presence of certain bacteria, such as Fusobacterium nucleatum, has been correlated with worse survival rates among CRC patients, highlighting its potential role as a biomarker and prognostic factor (37).

### 2.3 Gut microbiota as predictors of therapeutic response

Therapeutic outcomes in CRC can also be influenced by gut microbiota composition (38). The advent of immunotherapy has raised interest in understanding how gut microbiota affects patients’ responses to treatments such as immune checkpoint inhibitors(ICIs) (39). Several studies have reported that patients with a diverse gut microbiota are more likely to respond positively to ICIs, with favorable outcomes correlated to higher microbial diversity (40, 41) (**Figure 1**). Conversely, patients with specific microbial signatures may have poorer responses to treatment (42). For example, a recent study indicated that low levels of the genus *Akkermansia* were associated with decreased efficacy of anti-PD-1 therapy in CRC patients (43). These findings suggest that gut microbiota profiling could potentially serve as a predictive biomarker for therapeutic responses, guiding personalized treatment strategies in CRC.

Despite the growing body of evidence supporting the role of gut microbiota in CRC, there are several gaps in our understanding that warrant further investigation (44). The mechanistic pathways through which specific gut microbes influence tumorigenesis and immune responses remain inadequately explored. Future studies should aim to delineate these pathways, potentially identifying novel therapeutic targets. Additionally, large-scale, multi-center studies are needed to establish a comprehensive understanding of gut microbiota’s role in CRC across diverse populations. Standardizing methodologies for microbiome profiling and integrating metagenomic, metabolomic, and immune profiling approaches will enrich our insights into the interactions between gut microbes and host biology (45).

In summary, gut microbiota plays a multifaceted role in CRC pathogenesis, impacting tumor immune surveillance and therapeutic responses (46). The evidence suggests that both the composition and activity of gut microbes can markedly influence tumor development and progression, as well as the efficacy of treatment modalities (47). Understanding these complexities opens new avenues for personalized medicine in CRC, as targeting gut microbiota or modulating their composition could form part of future therapeutic strategies. Continued research in this field is essential for translating these findings into clinical practice, ultimately aiming to improve outcomes for CRC patients.

## 3 Gut microbiota and tumor immune surveillance

**Figure 2:**
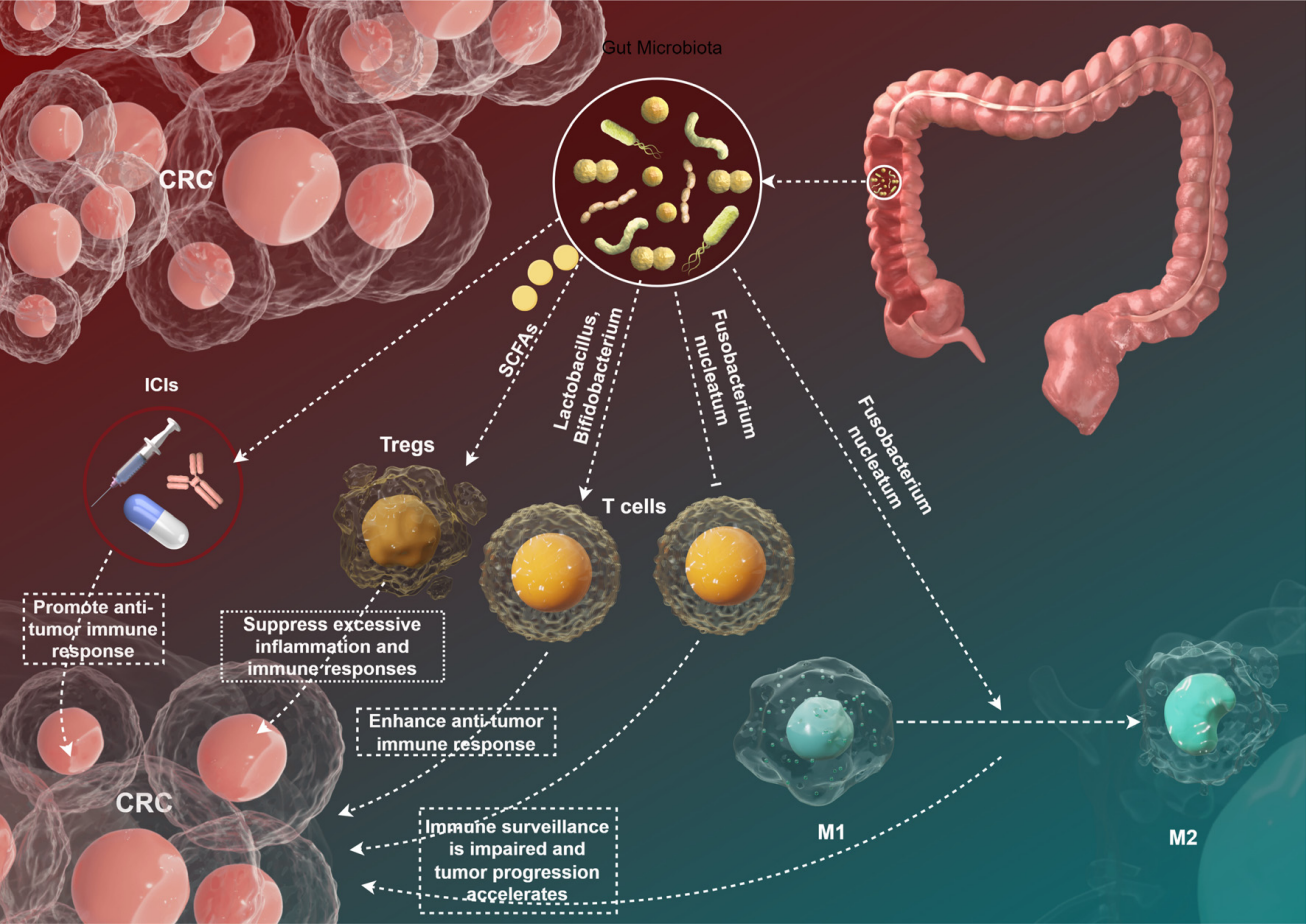
This figure to show the gut microbiota and tumor immune surveillance in CRC by Figdraw.

The intricate relationship between gut microbiota and the immune system has garnered significant attention in recent years, particularly regarding its implications for tumor immune surveillance in CRC. The gut microbiota contributes to the maintenance of immune homeostasis and the modulation of inflammatory responses, thus influencing the development and progression of malignancies (**Figure 2**). In this section, we will explore the current understanding of how gut microbiota interacts with tumor immune surveillance mechanisms, emphasizing various microbial species, their functional roles, and the diverse outcomes on tumor immunity.

### 3.1 Microbial composition and immune modulation

The composition of gut microbiota varies significantly among individuals, influenced by factors such as diet, lifestyle, and genetic background (6). Specific microbial communities have been shown to have distinct effects on host immunity. For example, a study by Luo et al. identified that a diverse gut microbiota composition is associated with enhanced anti-tumor immunity in CRC mouse models (48). The presence of beneficial bacteria, such as *Lactobacillus* and *Bifidobacterium*, was correlated with the activation of T cells and the production of pro-inflammatory cytokines that are crucial for anti-tumor responses (49) (**Figure 2**). Conversely, dysbiosis—characterized by an imbalance in microbial populations—has been linked to impaired immune surveillance and increased cancer risk (50). The pathogenic bacterium *Fusobacterium nucleatum* has been implicated in promoting CRC through multiple mechanisms, including the alteration of immune cell function. *Fusobacterium nucleatum* can inhibit the activity of cytotoxic T lymphocytes and promote the polarization of macrophages toward an M2 phenotype, which is associated with immunosuppression and tumor progression (51) (**Figure 2**).

### 3.2 Impact of gut microbiota on immune cell dynamics

Gut microbiota interact with various immune cell types, including dendritic cells (DCs), T cells, and regulatory T cells (Tregs), ultimately influencing the tumor microenvironment (52) (**Figure 2**). SCFAs, particularly butyrate, have been shown to enhance the differentiation and function of Tregs, promoting an anti-inflammatory environment (53). Arpaia et al. (54) demonstrated that butyrate plays a crucial role in Tregs differentiation, which can limit excessive inflammation and immune reactions against tumor cells. However, the role of Tregs in CRC is context-dependent; while they can prevent autoimmunity, their presence within tumors can also facilitate immune evasion by inhibiting effector T cell responses (54). Moreover, microbial metabolites can influence the maturation of dendritic cells, which are pivotal for T cell activation (55). Sivan et al. (56) reported that specific gut microbiota profiles could enhance the efficacy of immune checkpoint inhibitors by priming DCs to enhance anti-tumor immune responses. This finding underscores the potential for microbiota manipulation as an adjuvant therapy in immunotherapeutic approaches (56).

### 3.3 Gut microbiota and immune checkpoint inhibitors

The advent of ICIs has revolutionized the treatment landscape for various cancers, including CRC (57). The efficacy of ICIs is, in part, influenced by the composition of gut microbiota (39). A pivotal study by Wang et al. demonstrated that patients with a diverse gut microbiota had improved therapeutic responses to PD-1 blockade compared to those exhibiting dysbiosis (58) (**Figure 2**). The authors found that the presence of specific microbial taxa, including *Akkermansia muciniphila* and *Faecalibacterium prausnitzii*, correlated with better clinical outcomes (58). The mechanisms underlying this phenomenon are thought to involve enhanced anti-tumor immunity facilitated by gut microbiota. Microbes can influence the systemic immune environment, potentially increasing the infiltration of effector T cells into tumors (59). Additionally, the production of SCFAs may contribute to enhanced responsiveness to ICIs by modulating local and systemic immune responses (60). However, the relationship between gut microbiota and ICI efficacy remains complex and necessitates deeper investigation. Some studies have reported conflicting results, indicating that certain microbial profiles may be associated with reduced effectiveness of ICIs (38). For example, Routy et al. (61) found that specific Enterobacteriaceae members might be linked to poor responses to anti-PD-1 therapy in advanced melanoma, highlighting the need for personalized approaches in microbiota-targeted therapies.

### 3.4 Microbial metabolites as mediators of cancer host immune response

In addition to their direct effects on immune cells, gut microbiota also influence cancer host immune responses through the production of various metabolites. These microbial metabolites act as mediators that can modulate immune cell function, inflammation, and tumor microenvironment, thereby affecting antitumor immunity (62). SCFAs, such as butyrate, acetate, and propionate, are produced by the fermentation of dietary fibers by commensal bacteria. SCFAs have been shown to exert anti-inflammatory effects and enhance immune responses against tumors. For instance, butyrate can promote the differentiation of Tregs, which play a crucial role in maintaining immune homeostasis and preventing excessive inflammation (63). Additionally, SCFAs can enhance the production of cytokines such as IL-10 and transforming growth factor-beta (TGF-β), which are essential for immune regulation (60).

Bile acids, which are derived from cholesterol metabolism, can also be modified by gut microbiota. Microbial bile acid metabolites have been shown to influence immune cell function and tumor progression. For example, certain bile acid metabolites can activate the aryl hydrocarbon receptor (AhR), which is involved in the regulation of immune responses and has been implicated in cancer development (64). Tryptophan metabolism by gut microbiota produces various metabolites, including indole-3-acetic acid and indole-3-propionic aci, which have been shown to modulate immune responses. These metabolites can influence the activity of immune cells such as dendritic cells and T cells, thereby affecting antitumor immunity (65). Other microbial metabolites, such as succinate and formate, have also been implicated in modulating immune responses. For example, succinate has been shown to enhance the production of pro-inflammatory cytokines such as IL-1β and tumor necrosis factor-alpha (TNF-α), which can promote antitumor immunity (66).

### 3.5 The impact of gut microbiota on humoral immunity in antitumor responses

In addition to its role in modulating cellular immunity, gut microbiota also significantly influences humoral immunity, which plays a crucial role in antitumor responses (67). Humoral immunity involves the production of antibodies by B cells, which can recognize and neutralize tumor antigens, thereby inhibiting tumor growth and metastasis. The gut microbiota can affect humoral immunity through multiple mechanisms, including the regulation of B cell development, antibody production, and the modulation of immune cell interactions (67). Gut microbiota plays a vital role in the development and maturation of B cells. Studies have shown that specific microbial communities can promote the differentiation of B cells into plasma cells, which are responsible for antibody production. For instance, segmented filamentous bacteria have been shown to induce the production of IgA antibodies in the gut, which can help maintain gut homeostasis and prevent the overgrowth of pathogenic bacteria (68). Similarly, certain bacteria, such as Bifidobacterium and Lactobacillus, can stimulate the production of IgG antibodies, which are crucial for neutralizing tumor antigens and enhancing antitumor immunity (69).

Gut microbiota can also modulate the interactions between immune cells, including B cells, T cells, and DCs, thereby influencing humoral immunity. For example, SCFAs produced by gut microbiota can enhance the activation and proliferation of B cells, leading to increased antibody production (70, 71). Additionally, SCFAs can modulate the function of DCs, which play a crucial role in antigen presentation and the activation of T cells. By enhancing the function of DCs, gut microbiota can indirectly influence humoral immunity by promoting the activation of T cells, which can help regulate B cell responses (72). The influence of gut microbiota on humoral immunity has significant implications for antitumor responses. Studies have shown that specific microbial communities can enhance the production of antibodies that target tumor antigens, thereby improving antitumor immunity (56, 73). For instance, a study demonstrated that the administration of probiotics could increase the production of IgG antibodies against tumor antigens in CRC patients, leading to enhanced antitumor effects (74). Similarly, another study found that the presence of certain bacteria, such as Lactobacillus rhamnosus, could enhance the production of antibodies that neutralize tumor antigens, thereby inhibiting tumor growth and metastasis (67).

## 4 Gut microbiota and therapeutic response in CRC

The role of gut microbiota in modulating therapeutic responses in CRC has emerged as a significant area of research (75). Beyond its established function in homeostasis and immunity, the gut microbiome is increasingly recognized for its influence on treatment efficacy, particularly in the context of chemotherapy, targeted therapy, and immunotherapy (47) (**Table 1**).

**Table 1:** Studies on the relationship between gut microbiota and treatment response of CRC.

Authors	Gut Microbiota Category/Intervention/target	Animal Model/Human Study	Mechanism of Action	Role in CRC	Reference
Chang et al. (2020)	FMT	Human Study	Prevented intestinal injury, modulated TLRs expression	Influenced chemotherapy tolerance	(76)
Ghosh et al. (2022)	Microbial Metabolites	Human Study	Sensitized colorectal tumors to 5-FU by modulating drug transporters	Inhibited colonic tumor progression	(77)
Wang et al. (2021)	Dysfunctional TGF-β signaling	Animal Model	Altered gut microbiome	Confferred resistance to 5-FU	(78)
Li et al. (2024)	*Fusobacterium nucleatum*	Human Study	Induced resistance to oxaliplatin	Inhibited ferroptosis	(79)
Dalmasso et al. (2024)	Colibactin-producing Escherichia coli	Human Study	Promoted EMT	Enhanced resistance to chemotherapy	(80)
Xiaofeng et al. (2023)	Gut Microbiota	Human Study	Correlated with leukopenia after chemotherapy	Predicted adverse drug reactions	(81)
Chen et al. (2022)	Nitroreductase-instructed supramolecular assemblies	Human Study	Regulated gut microbiome	Enhanced CRC treatments	(82)
Hamidi Nia and Claesen (2022)	Engineered Cancer Targeting Microbes	Human Study	Delivered therapeutic agents directly to tumor site	Potential tool in CRC therapy	(83)
Lamaudière et al. (2023)	CRC Gut Environment	Human Study	Promoted multidrug-resistant phenotype	Hindered treatment efficacy	(84)
Liu et al. (2023)	Indole Metabolites	Human Study	Mediating host immune responses	Altered therapeutic effects	(65)
Kikuchi et al. (2020)	Tumor-Infiltrating Immune Cells	Human Study	Correlated microbiota composition with immune cell density	Enhanced infiltration of effector immune cells	(85)
Montalban-Arques et al. (2021)	Commensal *Clostridiales Strains*	Animal Model	Mediated effective immune responses	Direct antitumor effects	(86)
Sui et al. (2020)	YYFZBJS	Animal Model	Remodeled gut microbiota	Inhibited Tregs generation, enhanced antitumor responses	(87)
Zhang et al. (2021)	Pectin Supplement	Animal Model	Enhanced anti-PD-1 efficacy	Integral role in immunotherapeutic responses	(88)
Huang et al. (2022)	FMT	Human Study	Synergistic effects with anti-PD-1 therapy	Enhanced therapeutic potential of ICIs	(89)
Owens et al. (2021)	*Lactobacillus rhamnosus*	Human Study	Orchestrated an antitumor immune response	Improved anti-tumor immunity	(67)
Bi et al. (2023)	Aryl Hydrocarbon Receptor Nuclear Translocator	Human Study	Regulated neutrophil recruitment	Influenced by gut microbiota	(64)
Ajab et al. (2024)	Microbiota Composition	Human Study	Effect on immunotherapy outcomes	Systematic review of microbiota-therapy interactions	(38)

FMT, fecal microbiota transplantation; TLRs, toll-like receptors; 5-FU, 5-fluorouracil; EMT, epithelial to mesenchymal transition; CRC, colorectal cancer; Tregs, regulatory T cells; YYFZBJS, Yi-Yi-Fu-Zi-Bai-Jiang-San; PD-1, programmed death factor-1; ICIs, immune checkpoint inhibitors.

### 4.1 Influence on chemotherapy response

The impact of gut microbiota on chemotherapy outcomes in CRC has garnered increasing attention in recent years. Emerging evidence suggests that the composition and functional capacity of the gut microbiome can significantly alter the effectiveness and tolerability of common chemotherapeutic agents, such as 5-fluorouracil (5-FU) and oxaliplatin. Chang et al. (76) demonstrated that FMT not only prevented intestinal injury associated with chemotherapy but also reduced the systemic toxic effects of 5-FU and oxaliplatin through the modulation of Toll-like receptor (TLR) expression. This indicates that a balanced gut microbiome may enhance the tolerance of chemotherapy, revealing a potential approach for improving patient outcomes.

However, while some studies report beneficial associations between specific microbial taxa and enhanced chemotherapy response, others present contrasting findings. For example, Ghosh et al. (77) reported that certain microbial metabolites can sensitize colorectal tumors to 5-FU by modulating drug transporters via the FOXO3-FOXM1 axis, thereby inhibiting colonic tumor progression. In contrast, Wang et al. (78) found that mice with dysfunctional TGF-β signaling developed an altered gut microbiome that conferred resistance to 5-FU, highlighting the complexity of interactions between host immune responses, microbiome composition, and therapeutic efficacy. This discrepancy suggests that variations in microbiome composition might yield divergent effects on chemotherapy sensitivity, underscoring the need for personalized therapeutic strategies.

Moreover, certain bacterial species have been implicated in chemotherapy resistance mechanisms. *Fusobacterium nucleatum*, for instance, has been shown to induce resistance to oxaliplatin by inhibiting ferroptosis through the E-cadherin/β-catenin/GPX4 pathway (79). Similarly, another study identified colibactin-producing *Escherichia coli* as promoting resistance to chemotherapy by inducing epithelial-to-mesenchymal transition (EMT) during tumor progression (80). These findings highlight the potential for certain gut bacteria to not only correlate with adverse chemotherapy outcomes but also actively mediate resistance, complicating the therapeutic landscape for CRC.

In addition to these species, modifications in the gut microbiome post-chemotherapy have been shown to correlate with clinical outcomes. Xiaofeng et al. (81) observed a significant correlation between shifts in the gut microbiota and instances of leukopenia following chemotherapy in CRC patients, suggesting that microbiome alterations may predict adverse drug reactions. Such insights stress the importance of monitoring microbiome composition not only as a potential predictive biomarker but also as a target for therapeutic intervention.

Collectively, the interplay between gut microbiota and chemotherapy in CRC illustrates a complex relationship that can influence both efficacy and toxicity of treatment. This necessitates a comprehensive understanding of microbiome dynamics in the context of CRC therapy, paving the way for strategies that incorporate microbiota modulation to enhance therapeutic outcomes and mitigate detrimental effects. Further research is imperative to elucidate these relationships and their underlying mechanisms, ultimately contributing to the development of more effective, personalized cancer treatments.

### 4.2 Role in targeted therapies

The interplay between gut microbiota and therapeutic responses in CRC is gaining recognition, particularly in the context of targeted therapies. Recent studies suggest that the gut microbiota can modulate the effectiveness of various targeted treatments, including immunotherapies. Chen et al. (82) demonstrated that nitroreductase-instructed supramolecular assemblies can enhance the efficacy of CRC treatments by regulating the gut microbiome, highlighting the potential of microbiota modulation to increase therapeutic success in resistant tumors. Conversely, there is a variability in responses based on individual microbial communities, which can differ significantly between patients, affecting treatment outcomes.

Moreover, engineered microbes have emerged as highly promising tools in CRC therapy. Hamidi Nia and Claesen (83) described the use of cancer-targeting microbes and encapsulation devices to deliver therapeutic agents directly to the tumor site by navigating through the gut microbiome. However, the variability in the gut microbiome composition among patients complicates the development of standardized microbial therapies, raising concerns about the reproducibility of results across diverse populations.

Furthermore, Lamaudière et al. (84) have indicated that the CRC gut environment can promote a multidrug-resistant phenotype of ESKAPE pathogens, which may hinder treatment efficacy. Additionally, the role of gut-derived metabolites, particularly indole metabolites from tryptophan metabolism, has been implicated in mediating host immune responses and altering therapeutic effects, emphasizing the complexity of host-microbiota interactions in treatment (65).

In summary, while evidence supports a significant role of microbiota in CRC targeted therapies, inter-individual variability and microbial community dynamics remain critical hurdles in translating these findings into clinical practice. Further investigations are needed to elucidate consistent mechanisms and optimize therapies based on microbiota profiling, ultimately enhancing patient outcomes in CRC management.

### 4.3 Immunotherapy and gut microbiota

The gut microbiota has emerged as a critical factor influencing immune responses and therapeutic outcomes in CRC immunotherapy. A growing body of research illustrates how various microbial populations can either promote or inhibit anticancer immune responses, thereby shaping the efficacy of treatments such as ICIs. For example, Kikuchi et al. (85) characterized tumor-infiltrating immune cells in CRC and revealed a correlation between microbiota composition and the density of specific immune populations, such as T cells, in the tumor microenvironment. This suggests that a favorable gut microbiota may enhance the infiltration of effector immune cells, thereby potentially improving therapeutic outcomes.

Moreover, certain bacterial strains have shown promise in augmenting anti-tumor immunity. Montalban-Arques et al. (86) demonstrated that commensal *Clostridiales strains* could mediate effective immune responses against solid tumors, indicating that specific gut microbiota can have direct antitumor effects. In contrast, other studies highlight the potential for microbial dysbiosis to impair effective immune surveillance. Sui et al. (87) reported that treatment with Yi-Yi-Fu-Zi-Bai-Jiang-San (YYFZBJS) in Apc(Min/+) mice resulted in gut microbiota remodeling, which, in turn, inhibited Tregs generation, thus enhancing anti-tumor responses. This duality in microbiota influence underscores the complexity of interactions between gut communities, immune regulation, and therapeutic efficacy.

The potential of gut microbiota to predict immunotherapy outcomes has also been emphasized in recent studies. Zhang et al. (88) found that pectin supplementation significantly enhanced the efficacy of anti-PD-1 therapy in tumor-bearing mice with humanized gut microbiota derived from CRC patients, highlighting the integral role of specific dietary components and gut microbiota in immunotherapeutic responses. Additionally, Huang et al. (89) reported that FMT combined with anti-PD-1 therapy exhibited synergistic effects, suggesting that modulation of gut microbiota can play a pivotal role in enhancing the therapeutic potential of ICIs.

However, some discrepancies exist within the literature regarding the role of specific microbial taxa in CRC immunotherapy. For instance, while some studies have linked the abundance of certain bacteria, such as *Lactobacillus rhamnosus*, with improved anti-tumor immunity (67), others suggest the involvement of different microbial populations in negatively impacting immune responses, thus complicating the landscape of microbiota-therapy interactions (38, 64). Furthermore, the role of the AhR nuclear translocator in regulating neutrophil recruitment in CRC was shown to be influenced by gut microbiota, but the interplay remains complex and context-dependent (64).

In conclusion, the gut microbiota significantly influences the immune landscape in CRC and the outcomes of immunotherapy. While progress has been made in understanding these interactions, variability in findings and the complexity of microbiota-host dynamics necessitate further research. More extensive clinical studies that incorporate multi-omics approaches and exhaustive microbiota profiling may provide deeper insights into harnessing gut microbiota for enhanced immunotherapeutic strategies in CRC. Through such synergistic efforts, personalized microbiome-based interventions may one day become a standard adjunct to immunotherapy, ultimately improving patient outcomes in CRC management.

## 5 Microbiota modulation strategies in CRC: current advances

The translation of microbiota modulation strategies from experimental models to clinical practice remains a critical challenge in CRC management (44). While emerging evidence highlights the therapeutic potential of probiotics, prebiotics, dietary interventions, and FMT, key translational gaps—such as standardized protocols, safety monitoring, and personalized approaches—must be addressed to optimize clinical utility (**Table 2**). Below, we critically evaluate these strategies with a focus on their practical implementation in CRC care.

**Table 2:** Clinical studies of gut microbiota in CRC.

Authors	Gut Microbiota Category/Intervention	Study Type	Number of Patients	Patient Type	Findings	Reference
Gao et al. (2015)	Probiotics	Clinical Trial	60	CRC patients	Significantly altered mucosa-associated microbiota, improved immune responses	(90)
Zaharuddin et al. (2019)	Probiotics	Randomized, Double-Blind, Placebo-Controlled Trial	52	Post-surgical CRC patients	Reduced post-surgical complications, role in immune modulation	(91)
McLeod et al. (2023)	Mediterranean diet, weight loss	Randomized Controlled Lifestyle Intervention	192	African American/Black adults with obesity	Varied outcomes in CRC prevention	(92)
Sheflin et al. (2017)	Diet	Dietary Intervention Study	29	CRC survivors	Altered bacterial metabolism	(93)
Sofi et al. (2019)	Diet	Diet Comparison Study	150	CRC patients	Differing impacts on gut microbiota diversity	(94)
Watanabe et al. (2020)	Diet	Correlation Study	223	Middle-aged Japanese adults	Correlation between dietary intake and tumorigenic bacteria prevalence	(95)
Xie et al. (2019)	Prebiotics	Prebiotic Intervention Study	140	Perioperative CRC patients	Positively influenced immunologic indicators, modified gut microbiota structures	(96)
Huang et al. (2023)	Probiotics	Clinical Trial	100	Postoperative CRC patients	Improved gastrointestinal health, alleviated chemotherapy-induced dysbiosis	(97)
Wang et al. (2021)	*Fusobacterium*	Phase Ib/II Clinical Trial	42	Metastatic CRC patients	Combination with toripalimab showed promising results in gut microbiome analysis	(98)
Gu et al. (2024)	*Lachnospiraceae*	Single-Arm, Phase II Study	15	Metastatic CRC patients with third or above line setting	Modifications in gut microbiota associated with improved T-cell regulation	(99)

### 5.1 Probiotics

Probiotics, primarily Lactobacillus and Bifidobacterium strains, have demonstrated immunomodulatory and anti-tumor effects in preclinical models. However, clinical translation requires careful consideration of strain specificity, dosing, and timing. Gao et al. (90) demonstrated that the administration of probiotics in CRC patients significantly altered mucosa-associated microbiota, resulting in improved immune responses potentially beneficial for cancer therapy. A randomized, double-blind, placebo-controlled trial by Zaharuddin et al. (91) found that probiotics could reduce post-surgical complications in CRC patients, suggesting a role in post-operative recovery and immune modulation. Conversely, the impact of probiotics does not always lead to uniform results. For example, the study by McLeod et al. focused on a Mediterranean diet supplemented with specific probiotics demonstrating varied outcomes in CRC prevention (92). This disparity could be attributed to the strains of probiotics used and individual patient microbiota composition, emphasizing the need for personalized probiotic interventions. Hence, while probiotics are promising, their efficacy may be influenced by host-specific factors and the complexity of the intestinal ecosystem.

The selection of appropriate doses and timing of probiotic administration is crucial. Studies have shown that high doses of probiotics (e.g., 10^9^-10^10^ CFU/day) may be necessary to achieve significant clinical effects (90). However, long-term safety monitoring is essential, as excessive probiotic use may lead to potential risks such as infection in immunocompromised patients. Future research should focus on defining standardized protocols for probiotic use, including optimal doses, timing, and duration of treatment, to maximize therapeutic benefits while minimizing potential risks.

### 5.2 Dietary interventions

Dietary interventions have emerged as significant determinants of gut microbiota composition, thereby influencing CRC risk. The study by Sheflin et al. (93) highlights how supplementation with rice bran and navy beans altered bacterial metabolism in CRC survivors, suggesting that these dietary components could beneficially reshape the gut microbiome. Similarly, Sofi et al. (94) reported differing impacts on gut microbiota diversity in patients following meat-based versus pesco-vegetarian diets. This suggests a robust interaction between diet, microbiota and CRC risk. In a contrasting perspective, Watanabe et al. (95) identified a correlation between specific dietary intakes and the prevalence of tumorigenic bacteria, highlighting that not all dietary changes yield positive microbiota outcomes. This inconsistency calls for rigorous investigation into specific dietary components and their mechanisms of action. As dietary interventions continue gaining traction, large-scale, long-term studies are warranted to establish causal relationships and optimize dietary recommendations for CRC patients.

Personalized dietary interventions should be considered based on individual patient characteristics, such as genetic background, gut microbiota composition, and comorbidities (100). Long-term safety monitoring is also necessary to assess potential adverse effects of dietary changes, such as nutritional deficiencies or gastrointestinal discomfort (101). Future research should focus on developing standardized dietary guidelines for CRC patients, incorporating specific dietary components and their optimal amounts to achieve the best clinical outcomes.

### 5.3 Prebiotics

Prebiotics, defined as non-digestible food components that promote the growth of beneficial gut microorganisms, have also shown significant promise in CRC management. Xie et al. (96) reported that prebiotic supplementation positively influenced immunologic indicators and modified gut microbiota structures in perioperative CRC patients. Their study indicates prebiotics’ ability to promote favorable immune responses that might enhance cancer treatment outcomes. Further supporting prebiotics, Huang et al. (97) found that postoperative probiotic administration, combined with prebiotics, significantly improved gastrointestinal health and alleviated chemotherapy-induced dysbiosis. Future research should focus on elucidating the mechanistic pathways by which prebiotics modulate gut microbiota and their interactions with immunologic responses in CRC.

The selection of appropriate prebiotic types and doses is essential. Common prebiotics include inulin, fructooligosaccharides, and galactooligosaccharides, each with different effects on gut microbiota. Studies have shown that daily doses of 5-10 grams of prebiotics may be effective in modulating gut microbiota (102). However, long-term safety monitoring is necessary to assess potential adverse effects, such as gastrointestinal bloating or diarrhea. Future research should focus on defining standardized protocols for prebiotic use, including optimal types, doses, and duration of treatment, to maximize therapeutic benefits while minimizing potential risks.

### 5.4 Combination therapies

Combination therapies involving conventional treatments and microbiota modulation strategies have also been investigated. The recent study combining regorafenib therapy with toripalimab showed promising results when analyzing the gut microbiome, underscoring the importance of microbiota in modulating therapeutic responses (98). Similarly, the open-label phase II clinical trial examining Quxie Capsules in conjunction with conventional therapies identified modifications in gut microbiota associated with improved T-cell regulation in metastatic CRC (99). However, the varying outcomes from these trials stress the complexity of the interactions within the microbiome and its multifaceted impact on cancer therapies. Moreover, Taylor et al. (103) questioned the consistency of microbial alterations across different treatment regimens, emphasizing the need for further stratified studies involving controlled variables.

The modulation of gut microbiota as a strategy in CRC management is a rapidly evolving field, showing great promise in enhancing therapeutic responses and immune surveillance. While probiotics, dietary interventions, and prebiotics each present individual merits, the complexity and variability of microbiota responses warrant a nuanced understanding of patient-specific factors. Future studies should aim to bridge the gaps in current evidence, focusing on standardized methodologies to define the optimal use of microbiota modulation. Personalized approaches, considering individual microbiomic compositions, diet, and treatment histories, will be pivotal to harnessing the full potential of microbiota modulation in CRC management.

## 6 The dilemma we face for the future

The gut microbiota’s intricate relationship with CRC presents a complex dilemma for future research and clinical practice. Our understanding of the microbiota’s role in tumor immune surveillance and therapeutic response is still evolving, with numerous challenges and questions remaining unanswered (44).

One of the primary challenges is the heterogeneity of the gut microbiota across populations. Studies such as those by Mignini et al. and Herlo et al. have highlighted the variability in microbiota composition between CRC patients and healthy individuals, suggesting potential differences in immune responses and treatment outcomes (22, 104). The heterogeneity introduces a dilemma in developing standardized microbiota-based therapies, as the microbial signatures associated with CRC can differ significantly among individuals and even among different tumor sites within the same patient (22). Another dilemma arises from the dynamic nature of the gut microbiota. As noted by Hu et al., the gut microbiota is influenced by various environmental and genetic factors, leading to changes in bacterial diversity and function (44). This dynamism complicates the prediction of treatment response and the development of personalized therapies based on microbiota profiles.

The interaction between the gut microbiota and systemic therapies, including chemotherapy and immunotherapy, is another area of dilemma. While some studies suggest that certain microbiota members can enhance the efficacy of these treatments, others indicate potential resistance induced by specific bacteria. For instance, the work by Mager et al. demonstrated that specific bacteria could enhance the response to checkpoint inhibitor immunotherapy, while Gao et al. found that Fusobacterium nucleatum could also enhance programmed cell death ligand 1 (PD-L1) blockade efficacy in CRC (105, 106). These findings suggest a dual role for the microbiota in therapy, which poses a dilemma in harnessing its benefits without exacerbating resistance.

The potential for microbiota-based biomarkers to predict therapeutic outcomes is a promising yet challenging area. As reviewed by Herlo et al., various bacterial species have been implicated as potential biomarkers for CRC diagnosis and prognosis, but the variability in microbiota composition and the lack of standardized testing protocols present significant hurdles (22). The dilemma here is to identify which microbial signatures are most reliable and how to integrate them into clinical practice without causing undue harm or delaying treatment.

Finally, the ethical and practical considerations of microbiota modulation are a dilemma for future research. FMT has shown promise in altering the gut microbiota to improve treatment outcomes, as suggested by studies like those by Routy et al. and Baruch et al. (107, 108). However, the long-term effects of FMT are not fully understood, and there are concerns about the potential transfer of pathogenic microbes and the ethical implications of using human fecal matter as a treatment.

In conclusion, the future of gut microbiota research in CRC is fraught with dilemmas. The heterogeneity of the microbiota, its dynamic nature, the complex interactions with systemic therapies, the challenge of developing reliable biomarkers, and the ethical considerations of microbiota modulation all require careful consideration. As we move forward, it is crucial to address these dilemmas through multidisciplinary collaboration, rigorous clinical trials, and a deeper understanding of the microbiota’s role in CRC pathophysiology.

## 7 Discussion

Numerous studies have identified distinct microbial signatures associated with CRC, emphasizing a shift towards pathogenic bacteria and a decrease in beneficial commensals within the gut microbiome of CRC patients (19, 46). Specifically, the overrepresentation of Fusobacterium nucleatum has been implicated in promoting tumorigenesis through inflammatory mediators, disrupting immune homeostasis, and modulating tumor microenvironment (109). Conversely, beneficial genera such as Lactobacillus and Bifidobacterium have been associated with protective effects against CRC due to their roles in reinforcing gut barrier function and modulating immune responses (49, 110).

The complex interactions between the gut microbiota and the tumor microenvironment, including tumor cells, immune cells, and stromal cells, play a crucial role in CRC development and progression. The gut microbiota can influence tumor growth and metastasis by modulating various components of the tumor microenvironment. For instance, certain bacterial species, such as Fusobacterium nucleatum, can promote tumor growth by inducing chronic inflammation and activating oncogenic signaling pathways, such as the Wnt/β-catenin pathway, which leads to increased cell proliferation and survival (26, 35). Additionally, microbial metabolites, such as SCFAs, can modulate the tumor microenvironment by enhancing anti-inflammatory responses and promoting the differentiation of Tregs, which may suppress antitumor immunity (60, 63).

Furthermore, the gut microbiota can influence the tumor stroma by altering the expression of extracellular matrix components and growth factors, thereby affecting tumor cell adhesion, migration, and invasion. For example, BFT that disrupts cell junctions of E-cadherin and β-catenin, leading to increased intestinal permeability and inflammation, which can promote tumor progression (32, 34). These interactions highlight the potential for targeting the gut microbiota to modulate the tumor microenvironment and develop new therapeutic strategies for CRC.

Emerging evidence supports the notion that gut microbiota influences tumor immune surveillance (111, 112). Bacterial metabolites, such as SCFAs produced by fermentation of dietary fibers, have been linked to enhanced anti-tumor immune responses through modulation of T cell differentiation and activation (60, 66). For instance, butyrate has been shown to promote Tregs differentiation, potentially skewing the immune response towards an immunosuppressive phenotype (63). However, the relationship between gut microbiota and immune responses in CRC remains complex and context-dependent. Recent findings indicate that certain bacteria can elicit pro-inflammatory responses that may aid tumor progression while simultaneously overcoming immune checkpoints (16, 113). These dual roles present a conundrum in deciphering whether specific microbial components can be harnessed for therapeutic gain without exacerbating tumor growth. Further mechanistic studies are required to unravel these interactions and ascertain how varying microbiota compositions can selectively augment or diminish immune responses in CRC.

The therapeutic potential of modulating the gut microbiota in CRC treatment is a rapidly evolving area of research (114). Microbiome-targeted therapies, including probiotics, prebiotics, and FMT, have emerged as promising adjuncts to conventional treatments, such as chemotherapy and immunotherapy (115). Some studies suggest that specific microbial profiles could predict patient responses to immune checkpoint inhibitors, with Bacteroides and Prevotella being associated with favorable outcomes (116, 117). However, the clinical application of these findings is hindered by several factors. First, the heterogeneous responses observed in different patient cohorts indicate that microbiome modulation is far from a one-size-fits-all approach. Additionally, the potential side effects of manipulating the microbiome raise concerns about the safety and feasibility of such interventions in clinical settings (118). Moreover, the ethical implications of microbiome research must be considered, particularly in terms of informing patients about the experimental nature of these therapies and the variability of individual responses (119).

The intricate relationship between gut microbiota and CRC underscores its pivotal role in tumor immune surveillance and therapeutic response. While numerous studies highlight the potential of microbiome modulation to enhance immunotherapeutic effects, disparities in findings necessitate cautious interpretation. Future investigations should emphasize large-scale, multi-center studies to elucidate specific microbial signatures and their functional mechanisms, ultimately guiding personalized treatment strategies that integrate microbiota profiling to optimize patient outcomes in CRC management.

## 8 Conclusion

In conclusion, the intricate relationship between gut microbiota and CRC underscores its pivotal role in tumor immune surveillance and therapeutic response. While numerous studies highlight the potential of microbiome modulation to enhance immunotherapeutic effects, disparities in findings necessitate cautious interpretation. Future studies should prioritize the development of standardized methodologies for microbiome profiling and the integration of multi-omics approaches to better understand the complex interactions between gut microbiota and CRC. These advancements will ultimately guide personalized treatment strategies that incorporate microbiota profiling, optimizing patient outcomes in CRC management.
